# Injury severity level and associated factors among road traffic accident victims attending emergency department of Tirunesh Beijing Hospital, Addis Ababa, Ethiopia: A cross sectional hospital-based study

**DOI:** 10.1371/journal.pone.0222793

**Published:** 2019-09-26

**Authors:** Rediet Fikru Gebresenbet, Anteneh Dirar Aliyu

**Affiliations:** 1 Department of Clinical Trial, Armauer Hansen Research Institute, Addis Ababa, Ethiopia; 2 Department of Public Health, Jimma University College of Public Health, Jimma, Ethiopia; University of British Columbia, CANADA

## Abstract

**Background:**

Road Traffic Accidents have become an enormous global public health problem killing approximately 1.25 million people and injuring 20 to 50 million others yearly. It is the 10th leading cause of death universally and the number one cause of mortality of the young population between the ages of 5 and 29. Only few studies have been conducted on the severity of road traffic injuries in Ethiopia hence the need for the study.

**Objective:**

To assess injury severity level and associated factors among road traffic accident victims.

**Methods:**

A cross-sectional study of patients involved in road traffic accident and attended Tirunesh Beijing hospital, Addis Ababa, Ethiopia. Victims were consecutively recruited until sample size (164) attained during the study period. Data collectors administered a structured questionnaire. The collected data was then entered and cleaned using Epi info and exported to IBM SPSS for statistical analysis. Independent factors associated with injury severity were assessed using bivariate and multivariate logistic regression.

**Results:**

A total of 164 road traffic injury victims were included to the study. Prevalence of severe injury accounted for 36.6% of cases. “can read and write” educational status OR 35.194(95% CI; 3.325–372.539), sustaining multiple injury OR 18.400(95% CI; 5.402–62.671), sustaining multiple injury type OR 6.955(95% CI; 1.716–28.185) and being transported by ambulance from the scene of accident OR 13.800(95% CI; 1.481–128.635) were the explanatory variables found to have a statistically significant association with severe injury.

**Conclusion:**

This study showed road traffic accident is predominantly affecting the economically active, male young population. Not a single victim received pre-hospital care, majority were extracted by bystanders and most used commercial vehicle to be transported to a health institution reflecting the need for improvements in pre-hospital emergency services and socio-economic related infrastructures.

## Introduction

Road Traffic Injury (RTI) is one type of injury that occurs secondary to a Road Traffic Accident (RTA) which is defined as “An accident which occurred or originated on a way or street open to public traffic; resulted in one or more persons being killed or injured, and at least one moving vehicle was involved. These accidents therefore include collisions between vehicles, between vehicles and pedestrians and between vehicles and animals or fixed obstacles”(p.1) [1].

Per the Global status report on road safety, RTAs have become a huge global public health problem killing nearly 1.25 million people and injuring 20 to 50 million others annually, which is based on an information gathered from 180 countries[2, 3]. RTI is the 10^th^leading cause of death globally. WHO predicts that RTIs will rise to become the 5^th^ leading cause of death by 2030. RTIs are one of the top three causes of death for people aged between 5 and 44 years, and the leading cause of death of young people between the ages of 5 and 29 years[3, 4].

Despite the presence of several diverse ways to judge the severity of a crash, there are no generally agreed-upon definitions. Police and hospitals classify severity of RTIs differently. Generating an internationally agreed common definition of “serious” injuries based on the severity indexes determined by medical staff will aid to better understand the consequences of RTAs and to monitor progress of road crashes[5]. Some studies have tried to address accident-injury severity using various methods like the mixed-logit model. By using a combination of frequency models and mixed logit model to determine severity proportions, it will aid transport agencies to better understand and work on possible safety enhancements for an overall roadway safety. The mixed logit model permits flexibility to address segment-specific heterogeneity that can be attributed to various factors like geometric, pavement, traffic and weather-related variables[6]. One study that used mixed-logit model tried to analyze the difference in driver-severity injury between two groups (single-vehicle accidents and multi-vehicle accidents). It used a ten-year accident data that involved trucks on rural highways. The result showed that a considerable level of difference exists on the driver-injury severity in multi-vehicle and single-vehicle accidents. Analysis of injury severity on two groups of accidents separately showed the complex interactions of several variables and the nature of truck-driver injury in a better way[7].

The highest burden of road traffic death rates are in low-income and middle-income countries[8]. Up to 5% Gross Domestic Product (GDP) economic loss is attributed to Road traffic deaths and injuries in low and middle-income countries [9, 10]. Africa has the highest RTI death rate per population, with pedestrians and other vulnerable road users suffering the most. RTIs are now a momentous problem for Sub Saharan Africa(SSA), causing nearly one third of deaths in the region[11].

Ethiopia is a country in SSA with the lowest but a growing motorization globally with a fleet size of 587,400 and a motorization rate of only 2 cars per 1000 people. The accident rate per vehicle-km is too high; 195.1 traffic accidents per 10,000 vehicles. [8, 12–14].

Although Ethiopia has been affected by plentiful problems related to road traffic safety; studies on road traffic accident in the country are limited. The available studies mainly address burden of RTA, types of vehicle involved in injury, severity of injury, situation of the victims during accident, body region injured by the accident, and other associated factors. Still, most of them failed to assess the association between the explanatory variables and outcome of the victim like severity [15–17]. In addition, most studies used secondary traffic police data and aren’t institutional/hospital based[18–20]. Further on, hospital based studies didn’t include pediatric age groups in the research which primes for the need of this study [21, 22]. Therefore, this study aimed to assess the severity of RTIs and their associated factors (host, vehicle, socio-economic environment and physical environment related factors) among victims visiting the Emergency department of Tirunesh Beijing Hospital (TBH) as sufficient data is valuable to tackle this drastically rising, human killing problem. This study showed the level of severe injury to be 36.6% and revealed some explanatory variables having association with injury severity.

## Materials and methods

### Study setting and period

The study was conducted from June 3 –August 13, 2018 in Tirunesh Beijing Hospital, one of the public hospitals of Addis Ababa city, Ethiopia. Addis Ababa is the capital city of Ethiopia and is not only the seat of the Ethiopian federal government but also for African Union and many other international organizations including United Nations World Economic Commission for Africa. The city encompasses a total population of 2,738,248 inhabitants with female predominance 52.4% [23, 24]. Tirunesh Beijing Hospital works under the Addis Ababa City Administration Health Bureau, and is located in Akaki Kality Sub-city, woreda 1. It is giving an integrated health services since March 5, 2012 for Akaki Kality Sub-City inhabitants, other neighboring Sub-City inhabitants (Nifas Silk Lafto and Bole) as well as the surrounding Oromia Region inhabitants. It gives in-patient, out-patient, and emergency clinical services in various departments[25].

### Study design

Institutional based cross-sectional study design was implemented.

### Source and study population

The source population comprised of all patients attending Emergency Departments (adult and pediatric) of Tirunesh Beijing hospital after sustaining a RTA whereas study population were all RTA victims visiting the Emergency Departments of TBH during the study period.

### Inclusion and exclusion criteria

Inclusion criteria- RTA victims of all gender and age group (adults and pediatrics) who attended the Emergency Departments of Tirunesh Beijing Hospital between June 3 and August 13, 2018 who volunteered to participate, irrespective of the injury severity level were included in the study.

Exclusion criteria- The following group of RTA victims with no accompanying relative or informant to consent for the study were excluded from the study; those under the age of 18, unconscious victims and mentally ill patients. In addition, victims who died on-site and arrived at the hospital dead are also not included.

### Sample size and sampling procedure

Sample size (n) was determined using the single population proportion formula with the following assumptions: level of confidence (α) was taken as 0.05; Zα/2 = 1.96, a 5% margin of error (d = 0.05), and a proportion of severe injury of 10.87% taken from a study conducted at Tikur Anbessa Specialized Teaching Hospital (TASTH)[26]. Accordingly, a total of 164 patients were included in the study.

Systematic sampling technique was proposed to select the 164 patients who sustained RTA fulfilling the inclusion criteria during the study period using K^th^ interval.

K=N/n;201/164=1.2≈1

Where, 201 is the average number of RTA victims in 3 months (June-August 2017) gathered from the emergency departments’ logbook. Therefore, RTA victims visiting emergency departments of TBH were consecutively selected until sample size was achieved during the study period.

This study used fair sample size (n = 164) and those patients were selected systematically which lets us generalize the findings to Tirunesh Beijing Hospital but not to the general population of Ethiopia, Addis Ababa.

### Variables

**Dependent variable–**Severity level of road traffic injury [Severe injury and Non-severe injury]

#### Independent variables

**Host related variables [Driver, pedestrian or passenger]** ❖ Age ❖ Sex ❖ Marital status ❖ Educational status ❖ Occupation ❖ Driver’s driving experience year ❖ Type of road user [Pedestrian, Driver, Passenger] ❖ Circumstance of victim during the accident ❖ Body region injured [Multiple injury] ❖ Type of injury[Multiple injury type]

**Vehicle related variables** ❖ Vehicle type ❖ Type of accident

**Physical environment related variables** ❖ Time of the day ❖ Weather condition ❖ Lightning

**Socioeconomic environment related variables** ❖ Mode of transport to a health facility ❖ Time taken to reach a health facility

### Data collection techniques and instruments

A pretested, structured, interviewer administered, questionnaire was used. The questionnaire was able to assess all the variables which was developed after reviewing some literatures including injury surveillance guideline [19, 20, 22, 26–30]. Quality assurance of data was achieved through a careful design of the questionnaire. The initial English version of the questionnaire was translated to Amharic and then back translated into English independently to check for consistency and semantic validity. Later, it was pre-tested on 5% of the sample size at Zewditu memorial hospital before the actual data collection. Kampala Trauma Score II was used to assess the severity of injury (Table 1)[31]. The key factors that are associated with an outcome of a RTA is well described with Haddon Matrix; it clarifies the occurrence of injuries in terms of (human/host, vehicle/vector, physical environment and socioeconomic environment) during the pre-crash, crash and post-crash phases [32]. Additionally, medical records of the victims were reviewed to fill the clinical related data. Four Health Officers were the data collectors while 2 BSC nurses with master’s degree in Emergency Medicine supervised the data collection.

**Table 1:** Description of Kampala Trauma Score II (KTS II).

Label	Description		Score
A	Age (in years)	5–55	1
		<5 or >55	0
B	Systolic Blood pressure on admission	More than 89 mmHg	2
		Between 89 and 50 mmHg	1
		Equal or below 49 mmHg	0
C	Respiratory rate on admission	0-29/min	2
		30+	1
		≤9/min	0
D	Neurological status	Alert	3
		Responds to verbal stimuli	2
		Responds to painful stimuli	1
		Unresponsive	0
E	Score for serious injuries	None	2
		One injury	1
		More than one injury	0
Total (A+B+C+D+E) = ——————————-			

Interpretation = Scores; 9–10: Mild injury, 7–8: Moderate injury, 6 or less (≤6): Severe injury

### Theoretical definition

**Khat**- is a plant native to the Horn of Africa and the Arabian Peninsula. Chewing of khat leaves releases chemicals that are structurally similar to amphetamine which is said to cause euphoria followed by depression[33].

### Operational definitions

- **Severe injury**–According to Kampala Trauma Score II, score of 6 or less were considered as severe while KTS of 7 and 8 as Moderate [31]. However, for this study any RTA related injury with KTS II score of 8 or less is named as Severe injury.
- **Non-severe injury**—According to Kampala Trauma Score II; KTS II of 9 and 10 were considered as mild [31]. For this study, mild injuries are named as Non-severe injury.

### Data entry, processing and analysis

The collected data was entered and cleaned using Epiinfo ^TM^ version 7.2.2.2 and then exported to IBM SPSS/ Statistical Package for the Social Sciences version 20 for statistical analysis. Descriptive statistics was used to summarize the data using tables. Bivariate logistic regression was used to explore the association of each independent variable with the dependent variable. Explanatory variables with P-value of < 0.25 were considered for multivariate logistic regression to control the effect of other confounders. Finally, the significance level was set at P<0.05.

### Ethical clearance

Primarily, Ethical clearance was obtained from the Institutional Ethical Review Committee of Santé Medical College followed by Addis Ababa Health Bureau. Finally, approval was gathered from TBH. An informed written consent was obtained from all conscious patients prior to the data collection. In cases where the victim is unconscious and non-communicative, mentally ill, or in cases of those under the age of 18, attendants or parents were requested to consent for the study. The study was undertaken maintaining the confidentiality and privacy of each respondent.

## Result

A total of 164 Road Traffic Accident victims were recruited for the study, they were interviewed after receiving their consent. The analysis of all individuals was achieved as the response rate turned out to be 100% with no incomplete response to the survey.

### Socio-demographic characteristics of respondents

Out of the 164 RTI study participants who visited the adult and pediatric emergency departments of Tirunesh Beijing Hospital between the months of June and August 2018, 122 (74.4%) were male with a male to female ratio of 2.9:1. The victims’ age ranged from 4 to 70 with the mean and standard deviation of 29.4 and 9.8 respectively. The majority of victims are between 21–40 years of age group, accounting for 122 (74.8%). Four RTA victims were children. Eighty one (49.7%) of the respondents were single followed by another majority who are in the ever-married group, 82(50.3%). Regarding the respondents’ educational status 57(35%) of them had reached till secondary school followed by 34 (20.9%) of the respondents who are educated till primary school. Eighty six (52.4%) respondents are employed. Majority; 146(89%) of the road traffic accidents occurred in Addis Ababa whereas the rest 11% occurred in Oromia region. (see Table 2)

**Table 2:** Socio-demographic characteristics of road traffic accident victims, June-August 2018, Tirunesh Beijing Hospital, Addis Ababa, Ethiopia.

Variables	Non-Severe injury	Severe injury	Frequency (n = 164)	Percent
**Sex**				
Male	74	48	122	74.4
Female	30	12	42	25.6
**Age**				
20 and below	10	10	20	12.3
21–40	81	41	122	74.8
41 and above	13	8	21	12.9
**Marital status**				
Single	52	29	81	49.7
Ever Married	52	30	82	50.3
**Educational status**				
Cannot read and write	7	8	15	9.2
Can read and write	8	16	24	14.7
Primary school	19	15	34	20.9
Secondary school	42	15	57	35.0
Higher education	28	5	33	20.2
**Occupation**				
Employed	60	26	86	52.4
Unemployed	8	6	14	8.5
Driver	11	10	21	12.8
Others	25	18	43	26.2
**Region of accident**				
Addis Ababa	91	55	146	89.0
Oromia	13	5	18	11.0

### Basic characteristics of victims based on Haddon’s matrix

#### Host related characteristics

According to the classification of the victims, the majority 89(54.6%) were passengers followed by pedestrians 42(25.8%). Regarding the circumstance of victims during the occurrence of the accident, 90 of them (55.2%) were passengers in a vehicle whereas from the pedestrians 24 (14.7%) were walking on the roadside while 18 (11%) were crossing the road. From the 32 victims that are drivers, 28(87.5%) had driver’s license, 3(9.4%) were driving vehicles that didn’t require driver’s license (bicycle and cart), and the last 1 individual was driving without a driver’s license. Majority (63.3%) of the drivers’ driving experience ranged between 1–5 years. 16(59.3%) drivers used seatbelt while driving whereas none of the passengers (adult) wore seatbelt during the accident. From the two child passengers, both were in a vehicle where child restraint/ seatbelt use isn’t applicable. None of bicycle/motorcycle drivers and passengers wore helmet. Regarding alcohol use and driving, 28(87.5%) of them denied driving while being drunk, while 4(12.5%) of them claimed alcohol use while driving. From the pedestrians 9(21.4%) of them used alcohol prior to the accident whereas the remaining 33(78.6%) claimed no use. Three (9.4%) drivers used khat while the rest 29(90.6%) clamed no use prior to the occurrence of the accident. None of the pedestrians claimed use of khat. One driver was using mobile phone while driving whereas no pedestrian claimed mobile phone use when the road traffic accident happened. Majority of the victims sustained injury over single anatomic region 98(59.8%) followed by multiple injury 66(40.2%). Close to 3 quarter of victims sustained single injury type 126(76.8%) (see Table 3).

**Table 3:** Host related characteristics of road traffic accident victims, June-August 2018, Tirunesh Beijing Hospital, Addis Ababa, Ethiopia.

Host related characteristics of victims	Non-Severe injury	Severe injury	Frequency (n = 164)	Percent
**Classification of victim**				
Pedestrian	24	18	42	25.8
Driver	16	16	32	19.6
Passenger	64	25	89	54.6
**Circumstance of victim**				
Crossing the road	8	10	18	11
Walking on the roadside	16	8	24	14.7
Passenger in a vehicle	64	26	90	55.2
Driving	16	15	31	19
**Driver's license**				
Yes	14	14	28	87.5
No	1	0	1	3.1
Not required	1	2	3	9.4
**Driving experience**				
1–5 Years	10	9	19	63.3
Above 5 Years	6	5	11	36.7
**Seatbelt use by drivers**				
No	1	1	2	7.4
Yes	7	9	16	59.3
Not applicable	5	4	9	33.3
**Seatbelt use by passengers**				
No	48	14	62	73.8
Not applicable	12	10	22	26.2
**Child restraint/ Seatbelt use**				
Not applicable	1	1	2	100
**Helmet use (Driver & Passenger)**				
No	6	2	8	100
**Alcohol use (Driver)**				
No	15	13	28	87.5
Yes	1	3	4	12.5
**Alcohol use (Pedestrian)**				
No	20	13	33	78.6
Yes	4	5	9	21.4
**Khat use (Driver)**				
No	15	14	29	90.6
Yes	1	2	3	9.4
**Khat use (Pedestrian)**				
No	24	17	41	100
**Mobile phone use (Driver)**				
No	16	15	31	96.9
Yes	0	1	1	3.1
**Mobile phone use (Pedestrian)**				
No	24	17	41	100
**Multiple injury**				
Yes	22	44	66	40.2
No	82	16	98	59.8
**Multiple injury type**				
Yes	8	30	38	23.2
No	96	30	126	76.8

#### Agent/Vehicle related characteristics

Four and above-wheeled vehicles were attributed to most of RTAs in this study;113(69.3%) followed by three-wheeled and two-wheeled vehicles respectively. Two vehicle collision was found to be the commonest mechanism of road traffic accident 57(35%) followed by rolling of vehicle with or without collision with a fixed object 52(31.9%) (see Table 4).

**Table 4:** Description of agent/vehicle related characteristics of road traffic accident victims, June-August 2018, Tirunesh Beijing Hospital, Addis Ababa, Ethiopia.

Vehicle related characteristics	Non-Severe injury	Severe injury	Frequency (n = 164)	Percent
**Vehicle type**				
Two-wheeled vehicle	10	7	17	10.4
Three-wheeled vehicle	19	14	33	20.2
≥Four-wheeled vehicle	75	38	113	69.3
**Mechanism of the accident**				
Collision between vehicle and pedestrian	25	17	42	25.8
Two vehicle collision	37	20	57	35
Rolling of vehicle with or w/out collision with a fixed object	35	17	52	31.9
Falling from moving vehicle	7	5	12	7.4

#### Physical environment related characteristics

Regarding time of accident, 77 road traffic accidents (47%) occurred within the time range of 16:00–23:59. Half of the accidents occurred in daylight; 83(50.6%). About the weather condition, 146(89%) road traffic accidents occurred in a not-raining state (see Table 5).

**Table 5:** Description of physical environment related characteristics of road traffic accident victims, June-August 2018, Tirunesh Beijing Hospital, Addis Ababa, Ethiopia.

Physical environment related characteristics	Non-Severe injury	Severe injury	Frequency (n = 164)	Percent
**Time of accident**				
00:00–07:59	16	7	23	14.0
08:00–15:59	47	17	64	39.0
16:00–23:59	41	36	77	47.0
**Lighting condition**				
Daylight	52	31	83	50.6
Dark with no streetlight	27	12	39	23.8
Dark with streetlight	8	10	18	11.0
Dusk/dawn	17	7	24	14.6
**Weather condition**				
Raining	11	7	18	11.0
Not raining	93	53	146	89.0

#### Socio-economic environment related characteristics

Out of 164 individuals who sustained road traffic accident 132 (80.5%) of them were extracted by bystanders followed by police 26(15.9%). Only six victims were extracted by health professionals. All the 164 victims didn’t receive any prehospital care. Majority of the victims used commercial vehicle to reach an initial health institution 66(40.2%) followed by a private vehicle 44 (26.8%). A good number of victims (146) reached to an initial health institution within one hour while for 18(11%) of them it took them more than 1 hour (see Table 6).

**Table 6:** Description of socioeconomic environment related characteristics of road traffic accident victims, June-August 2018, Tirunesh Beijing Hospital, Addis Ababa, Ethiopia.

Socioeconomic environment related characteristics	Non-Severe injury	Severe injury	Frequency	Percent
**Victim is extracted by**				
Health professionals	1	5	6	3.7
Bystanders	85	47	132	80.5
Police	18	8	26	15.9
**Prehospital care**				
No	104	60	164	100.0
**Transport used from the scene**				
Ambulance	16	15	31	18.9
Commercial vehicle	46	20	66	40.2
Private vehicle	24	20	44	26.8
Other modes	18	5	23	14.0
**Time to reach initial health facility**				
1 Hour or less	99	47	146	89.0
More than 1 Hour	5	13	18	11.0

### Outcome of road traffic accident victims

According to Kampala Trauma Score II (KTS II) classification of trauma severity, out of the 164 road traffic accident victims, the majority of patients sustained mild injury104(63.4%). Moderate and severe injuries were 45(27.4%) and 15(9.1%) respectively. Nevertheless, for this study purpose it will be categorized as severe and non-severe injury;104 (63.4%) of them sustained non-severe injury (Mild) while 60 (36.6%) of them sustained severe injury (Moderate and Severe). 160 victims survived the accident whereas 4 individuals died during the course of treatment.

### Factors associated with injury severity

Injury severity level as a result of RTA had a statistically significant association with educational status, injured body part, injury type and mode of transportation used from the scene (Table 7).

**Table 7:** Bivariate and Multivariable analyses of predictors of road traffic accident victims’ injury severity levels, June-August 2018, Tirunesh Beijing Hospital, Addis Ababa, Ethiopia.

Variable	Categories	Injury severity level		COR 95% CI	AOR 95% CI
		Non-severe	Severe		
Education status	Cannot read and write	7	8	6.400(1.593–25.717)***	6.731(.528–85.845)
	Can read and write	8	16	11.200(3.129–40.084)***	35.194(3.325–372.539)***
	Primary school	19	15	4.421(1.375–14.213)**	3.235(.537–19.473)
	Secondary school	42	15	2.000(.653–6.126)*	2.145(.424–10.856)
	Higher education(Ref)	28	5	1***	1
Multiple injury	Yes	22	44	10.250(4.886–21.502)***	18.400(5.402–62.671)***
	No	82	16	1	1
Multiple injury type	Yes	8	30	12.000(4.972–28.962)***	6.955(1.716–28.185)***
	No	96	30	1	1
Mode of transport to initial health center	Ambulance	16	15	3.375(1.001–11.383)**	13.800(1.481–128.635)**
	Commercial vehicle	46	20	1.565(.510–4.803)	3.223(.421–24.688)
	Private vehicle	24	20	3.000(.945–9.521)*	6.712(.794–56.740)
	Other modes(Ref)	18	5	1*	1

*P<0.25

**P<0.05

***P<0.01

The odds of developing severe injury following a road traffic accident for those respondents who can read and write is 35.2 times than those who reached higher education level, AOR 35.2 (95% CI; 3.325–372.539). In addition, the odds of developing severe injury following a road traffic accident for those respondents who can read and write is 5.2 times than those who can’t read and write, 10.9 times than those educated till primary school and 16.4 times than those who reached secondary school level of education.

The odds of developing severe injury for victims who sustained multiple injury is 18.4 times than their counterparts, AOR 18.4(95% CI; 5.402–62.671).

Victims who sustained multiple injury type were nearly seven times more likely to develop severe injury than those with a single injury type, AOR 6.95(95% CI;1.716–28.185).

The odds of developing severe injury for accidents that occurred in the time range of 08:00–15:59 were found to be 58.8% lower than those accidents that occurred in the time range of 16:00–23:59 as per the bivariate analysis, COR 0.4(95% CI; 0.202–0.840) however this association wasn’t found to be statistically significant in the multivariable analysis after the control of potential covariates, AOR 0.3(95% CI 0.050–1.784).

Mode of transportation to an initial health institution have a statistically significant association with road traffic accident injury severity in this study. Victims who are transported by ambulance from the scene of the accident were approximately fourteen times more likely to be severely injured than those transported by other modes of transportation with AOR 13.8(95% CI; 1.481–128.635). In addition, victims transported by ambulance from the scene of the accident were 4.3 times more likely to have severe injury than those transported by commercial vehicles, and again those transported by ambulance were 2.1 times more likely to develop severe injury when compared with those transported by private vehicle from the scene of the accident.

Time to reach the initial health institution from the scene of the accident had association with road traffic injury severity in the bivariate analysis. The odds of developing severe injury is 5.5 times higher for victims who reached the initial health institution after an hour since the occurrence of the road traffic accident than those reaching in an hour or less, COR 5.5(95% CI; 1.845–16.261) but this association failed to be statistically significant in the multivariable analysis when potential confounders were controlled AOR 1.7 (95% CI; 0.372–8.015).

## Discussion

This study focused on injury severity level and associated factors among RTA victims attending the emergency departments of TBH, Addis Ababa, Ethiopia. Concerning age distribution of the victims, most (74.8%) were in their economically productive age group (21-40years). This is in line with the findings of Tanzanian, Kenyan and Ethiopian studies which show that RTA is frequently affecting the financially fruitful age group [26, 34, 35].

With regard to sex, male 122(74.4%) were more commonly affected by RTA than female 42 (25.6%) with a male to female ratio of 2.9:1. Male predominance in traffic accident is also reported by many other researches[22, 30, 36]. Seventy three percent of world’s road traffic fatalities are male[37]. Economically active young adults, particularly males, have been identified as high-risk group to be affected by this type of injury[29]. The reason for the young male predominance in road traffic accident might be attributed to the social acceptance of high levels of alcohol consumption by males, their involvement in laborious occupations and business activities that requires them to move from place to place.

Regarding educational status of the RTI victims, most were literate and reached secondary school57(35%). The odds of developing severe injury following a RTA for those respondents whose educational level is “can read and write” was found to be 35.2x higher than those who reached higher education level, OR 35.2 (95% CI; 3.325–372.539), 10.9 times than those educated till primary school and 16.4 times than those who reached secondary school level of education. According to a study done in Iran, the uneducated/ lowly educated victims suffered more fatal injury[38].

In this study, passengers were found to be frequently affected by RTA 89(54.6%) followed by pedestrians 42(25.8%). This finding is similar to a study done in Thika district hospital, Kenya and Arba Minch General hospital, Ethiopia [16, 35]. This might be attributed to the occurrence of repeated mass causalities occurring in city mini-buses which harbor twelve or more individuals in them, increasing the number of passenger victims to the emergency departments of the hospital in addition to vulnerability of passengers in developing countries. The most vulnerable road users in developing countries like Kenya, Ethiopia or Malawi, are pedestrians, passengers in informal, privately owned buses and minibuses, and bicyclists [39].

Majority of the drivers involved in collision in this study had driver’s license (87.5%) with most of the drivers’ experience year ranging between 1–5 years (63.3%). This result is similar with a study done in central Ethiopia[19].

Ethiopian government has enforced national preventive laws like speed limits, drink- driving law, motorcycle helmet law, seatbelt law, child restraint law, and banning of use of mobile phones while driving[40]. Despite these laws, this study revealed that no passenger fastened their seatbelts and only 59.3% of drivers used seat belt. This low figure is somewhat close to a previously mentioned study[30]. This might be due to the lack of seatbelts for passengers in many cars and commercial vehicles. Three wheeled vehicles like bajajs don’t have seatbelts even for the drivers. Regarding the use of helmets, they weren’t applied by any of drivers or passengers. There were 2 child passengers in this study where both were in vehicles that child restraint/ seatbelt use was not applicable because of the type of the vehicle. Underutilization of helmet by both drivers and passengers in this study might be due to the inadequate enforcement of the country’s national road safety laws and low public awareness on the protective effect helmets from severe injuries.

Drink-driving was witnessed in 12.5% of drivers and 21.4% of pedestrians were under the influence of alcohol during the occurrence of the accident. According to a study conducted in Kuala Lumpur, Malaysia, 20% of fatal pedestrians were positive for alcohol. The study highlighted the alarming problem of drink-and-walk in the area[41]. Most surveys showed that those who drank alcohol; mainly drivers, were more likely to be injured by a road traffic accident [22, 34]. Relationship between alcohol use and severe injury was not observed in this study.

Only one driver claimed to have used khat whereas none of the pedestrians in this study claimed khat use. Similarly, only a single driver confessed to have been using mobile phone while driving whereas none of the pedestrians stated cellphone use during the occurrence of the road traffic accident. This figure of phoning especially while driving is low when compared to a study done on crash injury severity in Ethiopia[42]. This might be due to false answers or smaller numbers of drivers in this survey.

Concerning anatomic region of injury and injury type, this study revealed that the odds of developing severe injury is 6.95 times higher for victims who sustained multiple injury type when compared with victims with single injury type, OR 6.95 (95% CI; 1.716–28.185). In addition, the odds of developing severe injury is 18.4 times higher for individuals who were injured at two or more body parts (multiple injury) than those individuals injured over a single anatomic region. OR 18.4(95% CI; 5.402–62.671). This is comparable with studies conducted in Ethiopia and Tanzania [30, 34].

Regarding vehicle related factors, majority of the RTAs in this study were attributed to vehicles with 4 or more wheels followed by the 3-wheeled vehicle; bajaj 33(20.2%). Likewise, car, mini bus and bus were frequently ascribed to be common vehicle types in RTAs of Addis Ababa[28]. Unlike some other studies on the subject matter that showed vehicle-pedestrian collision to be the most common mechanism of RTA, in this research two-vehicle collision was found to be the commonest mechanism 57(35%) followed by rolling of vehicle 52(31.9%)[26, 30].

Regarding time of accident, 77 road traffic accidents (47%) occurred within the time range of 16:00–23:59 and half of the accidents occurred in daylight; 83(50.6%). This is similar with the Addis Ababa–Hawassa highway study and the large scale study conducted in the city of Addis Ababa[28, 42]. As most other surveys, majority 146(89%) of RTAs occurred in a not-raining weather condition [19, 43].

Even though pre-hospital care is said to be an important factor for minimizing the level of injury severity, this study revealed that none of the victims received some sort of health assistance at the crash scene. Similarly none received pre-hospital care according to a study conducted in TASH, and only 2% of victims received some sort of management in the Tanzanian study[26, 34].

Most of the victims were extracted by bystanders 132(80.5%) and majority used commercial vehicle to reach to a health institution; 66(40.2%) and only 18.9% of them were transported by ambulance. This finding is harmonized with these studies[26, 30, 35].

This study showed that, victims who were transported by ambulance from the scene of the accident were approximately fourteen times more likely to be severe than those transported by other modes of transportation with OR 13.8(95% CI; 1.481–128.635), 4.3 times more likely to be severe than those transported by commercial vehicles, and 2.1 times more likely to be severe when compared with those transported by private vehicle from the scene of the accident. To rationalize this finding, one might assume traveling by ambulance by itself can carry a risk for worsening injury severity as a result of high speeds of travel and possible lack of restraint. According to WHO report on road traffic injury prevention, there are risks associated with ambulance transport, both for those being transported by the ambulance as well as pedestrians. Safety standards such as child restraints and seatbelts are recommended [44].

The time frame which immediately follows an injury is a crucial period where life sustaining care should be initiated. The initiation of prompt management in this window allows the victim to have utmost chance of survival and minimizes further complications that might result in death[45]. As per this study, majority of the road traffic accident victims146(89%) arrived the initial health institute within the golden hour.

This survey revealed that according to KTS II scoring, majority of RTA victims sustained mild injury (63.4%) followed by moderate (27.4%) and severe injury (9.1%). This finding is close with a study conducted in Tikur Anbessa Specialized Teaching Hospital (TASH), where51.74% of victims sustained mild injury, 37.39% moderate injury and the recorded prevalence for severe injury was 10.87%[26].

This study comes with various limitations. To begin with it used small sample size which may not detect some associations. Furthermore, some variables were collected using self-report which has a probability of creating social desirability bias. For example, some driver respondents might reply ‘No’ to alcohol use, Khat use or mobile phone use and ‘Yes’ to seat belt use. Finally, some variables that might have an effect on injury severity like vehicle speed and road type were not assessed in this study.

## Conclusions

One hundred sixty four victims were included in this study. Majority of the RTAs occurred in Addis Ababa city. Young male individuals that are in their economically active age group were predominantly affected by road traffic accident. Most of them were in the ever-married group and reached secondary school education. Those victims in the “can read and write” group were more likely to be injured severely. Passengers were found to be frequently affected by RTA.

Sustaining injury at multiple body parts is associated with severe injury as well as victims who sustained two or more types of injury (multiple injury type) had higher odds of developing severe injury when compared with those with single injury type.

None of the passengers in this study fastened their seatbelt and helmet was not worn by any of the bicycle/motorcycle drivers and passengers. Alcohol and khat use were reported by only few respondents.

Most of the accidents were attributed to 4 and above wheeled vehicles and occurred in the late afternoon to mid-night time period, often in a daylight with a not-raining weather condition. Two-vehicle collision was found to be the commonest mechanism of accident.

Socio-economic related infrastructures were witnessed to be poor according to this research i.e. not a single person received pre-hospital care, majority of the victims were extracted by bystanders from the scene of the accident and most used commercial vehicle to go to a health institution. Only a few were transported by ambulance. Those victims, reaching a health institution by ambulance had a higher odds of developing severe injury when compared with those that arrive at health care systems in other modes. Majority of the victims reached an initial health institution within the golden hour.

There is an urgent need to improve post- trauma care, as socio-economic infrastructures are witnessed to be deprived in the area. The Federal Ministry of Health of Ethiopia should work on training of paramedics, police officers/ traffic police and if possible, the general public on basic first-aid skills. There needs to be an improvement in availing ambulances with proper quality i.e. equipping them with child restraint, adult seatbelts, first-aid materials, emergency drugs and qualified health professionals. For future researchers on the subject matter, it is recommended to include few more factors like road type and vehicle speed. In addition, analysis models such as Bayesian binary logit and mixed logit models could be used to assess factors related to injury severity level. Mixed logit models are said to better approximate to any degree of accuracy when compared to the standard logit model[46]. The following studies used these models which can be used as a reference for future study in the subject[47–49].

## Supporting information

S1 Appendix RTI severity questionnaire (English and Amharic versions). (DOCX)

S1 Dataset RTI questionnaire. (SAV)

## Abbreviations

BSC: Bachelor of Science
GDP: Gross Domestic Product
KTS II: Kampala Trauma Score II
RTA: Road Traffic Accident
RTI: Road Traffic Injury
SPSS: Statistical Package for social Science
SSA: Sub Saharan Africa
TASTH: Tikur Anbessa Specialized Teaching Hospital
TBH: Tirunesh Beijing Hospital
WHO: World Health Organization
