# Supplementary material for: Injury severity level and associated factors among road traffic accident victims attending emergency department of Tirunesh Beijing Hospital, Addis Ababa, Ethiopia: A cross sectional hospital-based study
Source: PLoS One. 2019 Sep 26;14(9):e0222793. doi: 10.1371/journal.pone.0222793 (PMC6762084; doi:10.1371/journal.pone.0222793)
Supplement: S1 Appendix — (DOCX) [file pone.0222793.s001.docx]

**English Version Questionnaire**

**Santé Medical College**

**Department of Public Health**

**Preamble**

Dear respondent! The purpose of this questionnaire is to collect data about the severity levels of Road Traffic Injury and its associated factors in Tirunesh Beijing Hospital. Questions included in this questionnaire enable the researcher to gather an information relating to the magnitude of Road Traffic injury in this hospital, victim’s injury severity level and possible factors which contribute for road traffic injury severity. The information you provide us will be kept strictly confidential. Your frank and genuine response to the questions is very vital in achieving goal of the study. Hence, I pleasantly ask your honest and non-biased cooperation for interview of this questionnaire.

**General instruction**: Choose the appropriate response that reflect victim’s situation and write the number of your choice in the corresponding provided box.

**Segment 1: Certification**

Interviewer’s Name: ________________________________________

Signature: ________________________________________

Date (D-M-Y) Gregorian calendar: ________________________________________

Field Supervisors Name: ________________________________________

Signature: ________________________________________

Name of the hospital: ________________________________________

Questionnaire ID Number
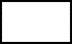

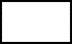

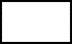


**Segment 2: Demographic and Socio-economic characters**

1. Sex (1= Male, 2= Female) ……………………………………………………….……….
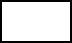


2. Age ……………………………………………………………………………….
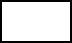

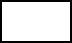


3. What is your marital status (1= Single, 2= Married, 3= Divorced, 4= Widowed) ………...
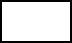


4. What is your educational status (1= Cannot read and write, 2= Can read and write, 3= Primary school, 4= Secondary school, 5= Higher education) …………………………….………….
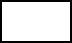


5. What is your occupation? (1= Merchant, 2= Driver, 3= Driver assistant, 4= Private employee, 5= Government employee, 6= Daily laborer, 7= Student, 8= Farmer, 9= Housewife, 10= Others)……………………………………………………………………………….……….
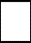

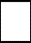


6. What was the region of the place where the accident took place? (1= Addis Ababa, 2= Oromia, 3= Others)……………………………………………………………………………..…….
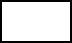


7. If the accident occurred in Addis Ababa, in which sub-city did the accident took place? (1= Addis Ketema, 2= Akaki Kality, 3= Arada, 4= Bole, 5= Gullele, 6= Kirkos, 7= Kolfe Keranio, 8= Lideta, 9= Nifas Silk-Lafto, 10= Yeka) ………………………………………..……
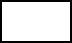

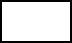


**Segment 3: Host related Characteristics**

**Instructions**: **-** Questions related to a driver will only be asked if the victim is the driver. If the victim is not a driver skip the question. The same instruction works for other types of victims (pedestrian and passenger) i.e.- passenger related questions should be asked only to passengers and pedestrian related questions should only be asked to pedestrians.

**–** Question number 18 and 19should be filled by the data collector and are not to be interviewed.

8. In which type you classify the victim? (1=Pedestrian, 2=Driver, 3= Passenger, 4= Other, 5= Unknown)…………………………………………………………...….………………..
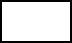


9. What was the circumstance of the victim during the accident? (1= Crossing the road, 2= Walking on the roadside, 3= Fell from a moving vehicle, 4= Passenger in a vehicle, 5= Driving, 6= Other, 7= Unknown)……………………………………………………………….……………….
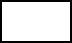


10. Does the driver have a driving license? (1=Yes, 2=No)…………...……………..……....
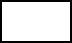


11. For how long have you been driving? …………………………………..……………….
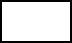


12. Was seat belt worn during the accident? (Driver and Passenger) (1=Yes, 2=No)………..
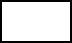


13. If the victim is a child was a child restraint or seatbelt used? (1= Yes, 2= No)…………...
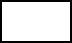


14. Is helmet used at the time of accident? (Driver and Passenger) (1=Yes, 2=No)……...…..
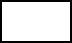


15. Did you use alcohol on the day of accident? (Driver and Pedestrian) (1=Yes, 2=No)…...
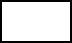


16. Did you use khat on the day of accident? (Driver and Pedestrian) (1=Yes, 2=No) ……....
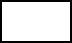


17. Was mobile phone used during the time of the accident? (Driver and Pedestrian) (1= Yes, 2= No)…………...…………………………………………………………………………..
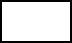


18. Which body part/ anatomic region of the victim is injured? (1=Head, 2=Neck, 3=Chest, 4=Abdomen, 5=Upper limb, 6=Lower limb, 7=Spine, 8=Maxillofacial, 9= Pelvis, 10= Multiple injury) ……………………………………………………………………….….
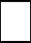

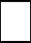

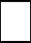

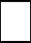

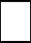

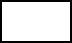


19. What is the type of injury sustained by the road traffic accident victim? (1= Soft tissue injury, 2= Fracture, 3= Thoracic injury, 4= Intracranial hemorrhage, 5= Abdominal visceral injury, 6= Pelvic injury, 7=Others,)………………………………………...............................
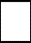

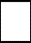

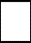

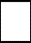


**Segment four: Agent related characteristics**

20. What was the type of vehicle that resulted in accident? (1=Motor cycle, 2= Bicycle, 3=Bajaj, 4= cart, 5= Car, 6=City mini bus, 7=Cross city mini bus, 8=Bus (city bus or long distance traveling), 9= Light truck (Pickup trucks) , 10= Heavy truck, 11=Truck with trailer, 12= Construction vehicle, 13=Others, 14= Unknown)…………………………...……….…
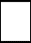

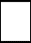


21. In which type of road traffic accident do you classify the accident? (1= Collision between vehicle and pedestrian, 2= Two vehicle collision, 3= Rolling of vehicle, 4=Collision between vehicle and animal, 5= Collision between vehicle and a fixed object, 6= Falling from moving vehicle, 7= Unknown)………………………………………………………….……………
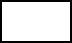


**Segment five: Physical environment related characteristics**

22. What was the time of accident? (1= 00:00-03:59, 2= 04:00- 07:59, 3= 08:00- 11:59, 4= 12:00-15:59, 5= 16:00- 19:59, 6= 20:00- 23:59, 7= Unknown)……………………………………..
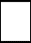

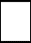


23. What was the lighting condition at the moment of accident? (1=Daylight, 2=Dark with no street light, 3=Dark with street light, 4= Dusk/dawn) ………………………………………...…...
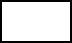


24. What was the weather condition during the time of accident at the scene of the crash? (1=Raining, 2=Not raining but slippery road, 3= Not raining and dry road) ………...…..…..
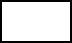


**Segment six: Socioeconomic environment related characteristics**

25. Did you receive a prehospital care? (1=Yes, 2=No)…………………………….…….....
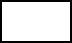


26. Who extract the victim from accident place? (1=Health professionals, 2=Bystanders, 3=Police, 4=Others) …………………………………………………………………………..……...
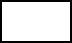


27. Which transport mode did you use to travel from the scene to an initial health facility? (1=Ambulance, 2= Commercial vehicle, 3=Police vehicle, 4= Private vehicle, 5= Carried by people, 6=Walking, 7=Others)……………………………………………………………
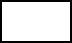


28. How long did it take to reach to an initial health facility? (1= 1 Hour or less, 2= More than 1 Hour)…………………………………………….………………………………………….
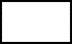


**Segment Seven: Treatment and outcome related data; to be filled strictly by the data collector**

29. What type of medical and surgical treatments were given to the road traffic accident victim? (1= Intravenous fluid, 2= Tetanus Antitoxin, 3= Blood transfusion, 4= Analgesics, 5= Antibiotics, 6= Wound cleaning and dressing, 7= Wound suturing, 8= Treatment of fracture, 9= Relocation of dislocation, 10= Under water seal drainage (Chest tube), 11= Craniotomy, 12= Burr hole, 13= Limb amputation, 14= Skin graft, 15= Exploratory laparotomy, 16= Other surgical procedures)…..…………………………………………………..……….
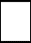

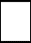

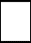

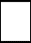

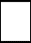

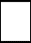

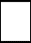

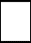


30. What is the severity of the injury based on KTS II (write the total score) …….………....
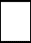

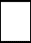


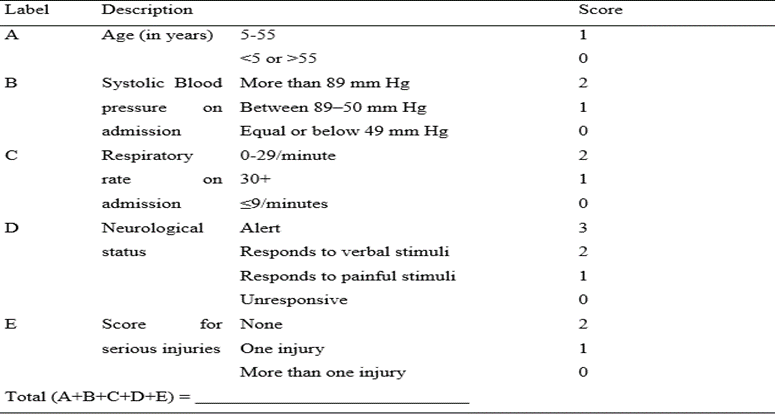


31. What is the general outcome of the patient? (1= Alive, 2= Dead) ……………………….
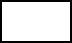
 32. What was the clinical outcome of the patient who survived the road traffic accident? (1= victim discharged well, 2= Victim discharged with a permanent disability, 3= Victim still under treatment during the period under the study, 4= victim is referred to other health facility, 5= Others)......……..…..………………………………………………………....………….
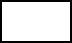


33. What is the length of hospital-stay of the victim? (Specify in days) ……………………..
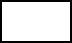


THANK YOU FOR YOUR COOPERATION

**የአማርኛ መጠይቅ ቅጽ**

**ሳንቴ ህክምና ኮሌጅ**

**የህብረተሰብ ጤና ሳይንስ ክፍል**

**መግቢያ**

ውድ ምላሽ ሰጪ! የዚህ መጠይቅ አላማ በጥሩነሽ ቤጂንግ ሆስፒታል አካባቢ የሚደርሱ የመንገድ ላይ የትራፊክ አደጋዎች የጉዳት መጠን በተመለከተ እና ተያያዥ ጉዳዮች ላይ መረጃ መሰብሰብ ነው፡፡ በዚህ መጠይቅ ላይ ያሉት ጥያቄዎቹ በዚህ ሆስፒታል የትራፊክ አደጋ ጉዳት መጠን እና ተጠቂዎቹ ያሉበትን ሁኔታ መመዘን ሲሆን የሚሰጡት መረጃ ሚስጥራዊነቱ የተጠበቀ ነው፡፡ እርስዎ የሚሰጡት እውነተኛ እና ታአማኒነት የተሞላበት ምላሽ ይህን ጥናት ለማሳካት ወሳኝ ሚና ይኖረዋል፡፡ ስለዚህ ከዚህ በመቀጠል በዚህ መጠይቅ ላይ በመሳተፍ ስለሚያደርጉልን ትብብር ምስጋናዬን እገልጻለሁኝ፡፡

**አጠቃላይ መግለጫ ፡-** የተጠቂን ሁኔታ የሚያሳይ: የሚመርጡን ቁጥር በተገለጸው ሳጥን ላይ ይጻፉ!

**ክፍል 1 ማረጋገጫ**

የቃለ መጠይቅ አድራጊ ስም

ፊርማ
ቀን/ወር/አመት እንደ እ.ኤ.አ

የመስክ ተቆጣጣሪ ስም፡

ፊርማ

የሆስፒታሉ ስም

የመጠይቁ መለያ ቁ.

**ክፍል፡2**

**የስነ ህዝብ እና ማህበራዊ ምጣኔ ሀብት ባህሪያት**

1. ጾታ፡ (1.ወንድ 2.ሴት)………………………………….……………..……………..
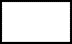

2. እድሜ…………………………………………………………...………………..……...
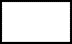

3. የጋብቻ ሁኔታ፡ (1. ያላገባ 2. ያገባ 3. የፈታ 4. የሙት ባለቤት………………...………..
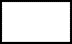

4. የትምህርት ደረጃዎ፡ (1. ማንበብ እና መፃፍ የማይችል 2. ማንበብ እና መፃፍ የሚችል 3. አንደኛ-ደረጃ 4. ሁለተኛ-ደረጃ 5. ከፍተኛ-ደረጃ)…...…………..................................
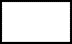

5. ስራዎ ምንድን ነው? 1. ነጋዴ 2. ሾፌር 3. የሾፌር ረዳት 4. የግል ሰራተኛ 5. የመንግስት ሰራተኛ 6. የቀን ሰራተኛ 7. ተማሪ 8. ገበሬ 9. የቤት እመቤት 10.ሌላ……………………………………………..……………………………
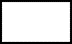

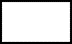

6. አደጋው የደረሰበት ክልል? 1. አዲስ አበባ 2.ኦሮሚያ 3. ሌላ………………………..…...
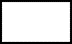

7. አደጋው አዲስ አበባ ውስጥ ከተከሰተ በየትኛው ክ/ከተማ ተከሰተ?
8. አዲስ ከተማ 2. አ/ቃሊቲ 3. አራዳ 4. ቦሌ 5. ጉለሌ 6. ቂርቆስ 7. ኮ/ቀራኒዮ 8. ልደታ 9. ን/ስ/ላፍቶ 10. የካ…….............………………
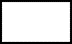

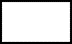


**ክፍል 3፡**

**ከምላሽ ሰጪ ጋር የተያዘ ባህሪያት**

**መግለጫ፡-**ከሾፌሮች ጋር የተያያዙ ጥያቄዎች ተጠቂው ሾፌር ከሆነ ብቻ ይጠይቃሉ፡፡ ሾፌር ካልሆነ ጥያቄው ይታለፍ፡፡ ለእግረኞች እና ተሳፋሪዎች ተመሳሳይ ትእዛዝ የሚሰራ ሲሆን ማለትም ከተሳፋሪ ጋር የተያዙ ጥያቄዎች ተሳፋሪውን ወይም ከእግረኛ ጋር በተያያዘ እግረኛን ይጠይቁ፡፡

ጥያቄ ቁጥር 18 እና 19 በመረጃ ሰብሳቢው መሞላት ሲኖርበት ቃለመጠይቅ አይኖረውም፡፡

1. በየትኛው ምድብ ተጠቂውን ይለያሉ?
2. እግረኛ 2. ሾፌር 3. ተሳፋሪ 4.ሌላ 5. አይታወቅም………………………....……...
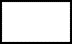

3. በአደጋው ወቅት የተጠቂው ሁኔታ ምን ይመስላል?
4. መንገድ እያቋረጡ 2. በመንገድ ዳር እየሄዱ 3. ከሚንቀሳቀስ ተሽከርካሪ ወድቀው 4. በተሽከርካሪ ውስጥ ያሉተሳፋሪ 5. እያሽከረከሩ 6. ሌላ 7. አይታወቅም…...…
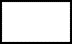


10. ሾፌሩ መንጃ ፈቃድ ነበራቸው? 1. አላቸው 2. የላቸውም…….……….…...
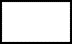


11. ለምን ያህል ጊዜ አሽከርክረዋል?……………………………………..........…………...……
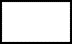


12. በአደጋው ወቅት የመቀመጫ ቀበቶ አድርገው ነበር? (ሾፌር እና ተሳፋሪ)

1. አድርገዋል 2. አላደረጉም…………………………………………………..………….....…
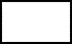


13. ተጠቂው ህፃን ከሆነ የህፃን ማሰሪያ ወይም የመቀመጫ ቀበቶ አድርጎ ነበር?

1. አድርገዋል 2.አላደረገም……………………………………………..………………..……
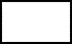


14. በአደጋው ወቅት የአደጋ ቀበቶ ተጠቅመዋል (ሾፌር/ተሳፋሪ) 1. አዎ 2.አይ………………..…...
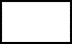


15. በአደጋው ወቅት አልኮል ተጠቅመዋል (ሾፌር/እግረኛ) 1.አዎ 2.አይ……………………….......
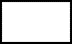


16. በአደጋው ወቅት ጫት ተጠቅመዋል (ሾፌር/እግረኛ) 1. አዎ 2.አይ…………………..…
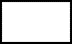


17. በአደጋው ወቅት ሞባይል ስልክ እየተጠቀሙ ነበር (ሾፌር/እግረኛ) 1. አዎ 2.አይ…….….
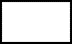


18. የተጎጅው የትኛው ሰውነት አካል ተጎድቷል 1. የራስቅል 2. አንገት 3.ደረት 4.ሆድእቃ 5.እጆች 6. እግሮች 7. የጀርባ አጥንት 8.መንጋጋ 9.የወገብ አጥንት 10. የበርካታ አካል ክፍል ጉዳት….
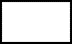

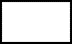


19. በተጎጂው ላይ ምን አይነት ጉዳት ደርሷል? 1. በስስ አካል ላይ ጉዳት 2. የአጥንት ስብራት 3. የደረት ጉዳት 4. የጭንቅላት ውስጥ ደም መቋጠር 5.የሆድ እቃ ላይ ጉዳት 6.የወገብ ጉዳት 7. ሌላ….………
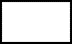


**ክፍል4፡-**

**ከተሽከርካሪው ጋር የተያያዙ ሁኔታዎች**

20. በአደጋው ወቅት የነበረው የተሽከርካሪው አይነት

1. ሞተር ሳይክል 2. ባይስክል 3. ባጃጅ 4.ጋሪ 5.መኪና 6.የከተማ ሚኒባስ 7.ሀገር አቋራጭ ሚኒባስ 8.ባስ/የከተማ ባስ ወይም የረጅም ርቀት ባስ 9.ቀላል ተሽከርካሪ/ፒክአፕ 10.ከባድ ተሽከርካሪ 11.ተሳቢያለው ተሽከርካሪ 12. የኮንስትራክሽን ተሽከርካሪ 13.ሌላ 14. አይታወቅም……………………..
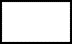

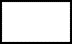


21. አደጋውን በየትኛው የመንገድ ላይ የትራፊክ አደጋ ይመድባሉ

1. በተሽከርካሪ እና ተጓዥ መካከል ግጭት 2. የሁለት ተሽከርካሪዎች ግጭት 3.የተሽከርካሪ መገልበጥ 4.በተሽከርካሪ እና እንስሳት መካከል ግጭት 5.በተሽከርካሪ እና ቋሚ ንብረት መካከል ግጭት 6.ከተሽከርካሪ መውደቅ 7. አይታወቅም …………………………………...................................................…
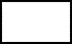


**ክፍል5 ፡-**

**ከአካባቢው ሁኔታ ጋር የተያያዙ ሁኔታዎች**

22. አደጋው የደረሰበ ትሰአት 1. 00፡00 – 03፡59 2.04፡00- 07፡59 3. 08፡00 -11፡59 4. 12፡00 -15፡59 5. 16፡00 - 19፡59 6.20፡00–23፡59 7.አይታወቅም….……………………………..
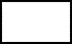


23. በአደጋው ወቅት የነበረው የብርሃን ሁኔታ? 1. ቀን 2. ምሽት የመንገድ ላይ መብራት የሌለበት 3. ጨለማ የመንገድ ላይ መብራት ያለበት 4. ጸሀይ ሲጠልቅ/ሲነጋ………………………..……...
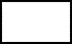


24. በአደጋው ወይም በግጭቱ ወቅት የነበረው የአየር ሁኔታ?1. እየዘነበ ነበር 2. ዝናብ የለም ሆኖም የሚያንሸራትት መንገድ ነበር 3. አይዘንብም ደረቅ መንገድ………………………….…….…..
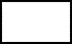


**ክፍል6፡-**

**ከሁኔታው ጋር የተያያዙ ማህበራዊ ምጣኔ ሀብት ሁኔታዎች**

25. ሆስፒታል ከመድረስዎ በፊት የቀድመ-ሆስፒታል የህክምና እርዳታ አግኝተዋል?

1. አዎ 2.አላገኘሁም………………………………...………………..…………….………...
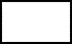


26. ከአደጋው ቦታ ተጎጅውን ማን አነሳ ? 1. የጤና ባለሙያ 2.አካባቢው የነበሩ ግለሰቦች 3. ፖሊስ 4.ሌላ………………………………………………….……………..…...………..
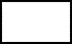


27. ከአደጋው ቦታ ወደ መጀመሪያው ጤና ተቋም ተጎጅውን ለመውሰድ ምንአይነት መጓጓዣ ተጠቅመዋል? 1. አምቡላንስ 2. የንግድ ተሽከርካሪ 3.የፖሊስ ተሽከርካሪ 4.ግል ተሽከርካሪ 5. ሰዎች ተሸክመው በማጓጓዝ 6. በእግር ጉዞ 7. ሌላ…………………………..…………..……
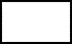


28. ወደ መጀመሪያው የጤና ተቋም ለመድረስ ምን ያህል ጊዜ ወሰደ?

1. 1 ሰአት ወይም ከዚያ በታች 2. ከ1 ሰአት በላይ……..…………………….……..………….
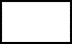


**ክፍል7፡-**

**ህክምና እና የውጤቱ መረጃ፣ በትክክል በመረጃ ሰብሳቢው መሞላት ያለበት!**

29. ለመንገድ ላይ የትራፊክ አደጋ ተጎጅ ምን አይነት የህክምና እና ቀዶ ህክምና እገዛ ተደርጓል?

1.ግሉኮስ 2.የቲታኖስ መድሃኒት 3.ደም ማዘዋወር 4.የህመም ማስታገሻ

5. አንቲባዮቲክ 6. ቁስል ማጽዳት እና ሽፈና 7. የቁስል መስፋት 8.የአጥንት ስብራት ህክምና 9.ውልቃት ማስተካከል 10.የደረት ላይ ቲዩብ ማስገባት 11.ክራዬቶሚ 12.በርሆል 13.የአጥንት ክፍል ቆርጦ ማውጣት 14.ቆዳ ከሰውነት ክፍል ላይ ወስዶ መለጠፍ 15. ኤክስፕሎራቶሪ ላፓራቶሚ 16.ሌላ የቀዶ ህክምና ስራዎች………………………………..………………………….…..…
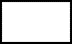

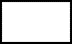


30. በኬቲኤስ 2 መሰረት የጉዳቱ ደረጃ/አጠቃላይ ውጤት ይጻፍ………………….……..….……..
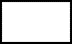


|  | **ዝርዝር** |  | **ውጤት** |
| --- | --- | --- | --- |
| ሀ | እድሜ /በአመት | 5-55  ከ5 በታች ወይም ከ55 በላይ | 1  0 |
| ለ | ሆስፒታል እንደደረሰ የላይኛው የደም-ግፊት መጠን | ከ89 ሚ.ሜ ሜርኩሪ በላይ  ከ89-50 ሚ.ሜ ሜርኩሪ  49 ሚ.ሜ ሜርኩሪ ወይም ከዚያ በታች | 2  1  0 |
| ሐ | ሆስፒታል እንደደረሰ የአተነፋፈስ ፍጆታ | 0-29/ደቂቃ  30+  ከ9/ ደቂቃ እኩል የሆነ ወይም በታች | 2  1  0 |
| መ | ራስን የማወቀ ብቃት | ንቁ  ለቃል ምላሽ ይሰጣሉ  ለህመም ስሜት ምላሽ አላቸው  ምላሽ የላቸውም | 3  2  1  0 |
| ሠ | የከባድ ጉዳቶች ውጤት | የለም  አንድ ጉዳት  ከአንድ ጉዳት በላይ | 2  1  0 |
| ድምር ሀ+ለ+ሐ+መ+ሠ= ___________________ | | | |

31. የታካሚው አጠቃላይ ውጤት ምንድን ነው?1. በህይወት አለ 2. አርፈዋል……………….….
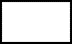


32. በህይወት የተረፍ የመንገድ ላይ ትራፊክ አደጋ የደረሰባቸው ግለሰብ የመጨረሻ ውጤት

1. በጤና ከሆስፒታል የወጡ 2. ቋሚ አካል ጉዳት የደረሰባቸው ና ከሆስፒታል የወጡ 3.በጥናቱ ጊዜ በህክምና ላይ ያሉ 4.ወደ ሌላ ጤና ተቋም ሪፈር የተደረጉ 5.ሌላ…………………………………………………………………………………...…….
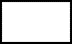


33.የተጎጂ የሆስፒታል ቆይታ ጊዜ? (በቀኖች ይገለጽ)……………………………………………..
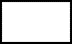


ለትብብሮ አመሰግናለሁ፡፡
